# Interplay of depressive symptoms and clinical oral health conditions: a systematic review and meta-analysis

**DOI:** 10.1590/1807-3107bor-2026.vol40.073

**Published:** 2026-09-18

**Authors:** Miguel Konradt MASCARENHAS, Marina da Costa ROCHA, Mariana Gonzalez CADEMARTORI, Marcos Britto CORREA, Francine dos Santos COSTA, Luiz Alexandre CHISINI, Flávio Fernando DEMARCO

**Affiliations:** (a) Universidade Federal de Pelotas – UFPel, School of Medicine, Graduate Program in Epidemiology, Pelotas, RS, Brazil.; (b) Universidade Federal de Pelotas – UFPel, School of Dentistry, Graduate Program in Dentistry, Pelotas, RS, Brazil.

**Keywords:** Depression, Dental Caries, Tooth Loss, Periodontal Diseases

## Abstract

This systematic review and meta-analysis investigated the potential links between clinically assessed oral health conditions and depressive symptoms in adults. Six databases were searched for observational studies examining dental caries, periodontal disease, or tooth loss through clinical examination, and depressive symptoms through validated instruments. Data extraction and quality assessment (JBI) were independently performed by two reviewers, and random-effects meta-analyses were conducted for compatible effect measures (OR), assessing heterogeneity. Out of 6.917 unique records, 20 studies were selected. The meta-analyses revealed increased odds of depression for patients who experienced tooth loss (pooled OR = 1.19, 95%CI: 1.09–1.30, I^2^ = 54.7%), supported by a dose-response gradient in subgroup analyses. Periodontal disease also increased the odds of depression (pooled OR = 1.59, 95%CI: 1.14–2.21, I^2^ = 0.0%). Conversely, depression as an exposure was associated with higher odds of dental caries (pooled OR = 1.20, 95%CI: 1.04–1.39, I^2^ = 0.0%), but not with periodontal disease. The relationship between oral and mental health seems to be condition-specific, with robust quantitative evidence indicating the importance of tooth loss in the development of depressive symptoms, which in turn increase the odds for dental caries, suggesting a possible feedback loop. Evidence for depression affecting periodontal status remains inconclusive.

## Introduction

Oral diseases are among the most prevalent chronic conditions globally, with dental caries and severe periodontitis affecting 35% and 11% of the global population,^1^ respectively, and tooth loss occurring in 4.4% of the world population.^2^ These conditions cause pain, functional limitations, and an overall lower quality of life.^3^ It has been estimated that about 5% of global health expenditure should be allocated to treating oral conditions.^4^

Concurrently, the prevalence of mental diseases has been increased worldwide in recent decades, with depression affecting 5% of the adult population globally.^5^ In Brazil, the prevalence of self-reported depression rose from 7.6% to 10.2% between 2013 and 2019.^6^

The potential association between oral health and depression has gained attention due to shared risk factors for both oral health outcomes and depression, such as economic disadvantage, education, and self-care behaviors.^7^ Some studies report depression as a risk factor for periodontal disease, dental caries, and tooth loss through behavioral and biological pathways,^8 9^ while others find no such associations.^10^ Similarly, emerging evidence suggests oral conditions, particularly tooth loss, may contribute to the development of depression through functional, aesthetic, and potentially neurobiological mechanisms,^11^ though findings remain inconsistent.^12^

Previous reviews have examined these relationships using self-reported measures of oral health conditions. Self-reports are prone to misclassification bias, particularly in individuals with depression. While a recent review attempted to update these associations, it has faced criticism for methodological limitations, such as applying cross-sectional risk of bias tools to longitudinal studies and overinterpreting causal links.^11^ Therefore, distinguishing between self-reported and clinically diagnosed conditions using rigorous methodological standards is crucial. This systematic review aims to advance the field by specifically synthesizing evidence on the interplay between depression and clinically assessed oral health conditions (dental caries, periodontal disease, and tooth loss) in adult populations.

## Methods

This systematic review followed the Preferred Reporting Items for Systematic Reviews and Meta-Analyses (PRISMA) 2020 guidelines.^13^ The primary research question was:

- Is there an association between depressive symptoms and clinically observed oral health conditions (caries, periodontal disease, and tooth loss) in the general adult population?

The goal of this review was to investigate both possible directions of the associations described above. This bidirectional approach is more clearly represented in the PECO (Population, Exposure, Comparison, Outcome) framework:

- **P:** Representative samples of the general adult or older adult population;
- **E:** Either (a) depressive symptoms assessed using validated psychometric instruments, or (b) clinically assessed oral health conditions;
- **C:** Individuals without the exposure of interest;
- **O:** The counterpart of the condition explored as exposure.

### Selection criteria

Studies were considered eligible if they: a) were original observational studies (cross-sectional, cohort, or case-control); b) included representative samples of the general adult population (≥ 18 years old); c) evaluated the association between depressive symptoms (assessed via validated psychometric instruments) and at least one clinically observed oral health condition (caries, periodontal disease, or tooth loss); and d) provided adjusted effect measures. Studies were excluded if they: a) recruited convenience samples or specific or restricted populations; b) relied solely on self-reported oral health measures; c) did not report validation of the depression assessment instrument; or d) were qualitative studies, reviews, editorials, or protocols. The protocol for this review was not registered in Prospero prior to the start of the study, which is acknowledged as a limitation.

### Search strategy

Six databases were consulted: BVS (Biblioteca Virtual em Saúde), PubMed, PsycInfo, SciElo, Scopus, and Web of Science. The initial searches were conducted between May and July 2025, with no restrictions on publication date or language. An updated search was performed in August 2025, but yielded no additional eligible studies.

The search strategies for each database are presented in Table 1.

**Table 1:** Search strategies for each database.

Database	Search terms		
BVS	((“Saúde Mental” *OR* “Mental Health”) *OR* (“Transtornos de Humor” *OR* “Mood disord*”) *OR* (“Transtornos Mentais Comuns” *OR* “Common Mental Disord*”) *OR* (“Depressão” *OR* “Depression” *OR* “Transtorno Depressivo” *OR* “Depressive Disord*” *OR* “Sintomas depressivos” *OR* “Sintomas de depressão” *OR* “Depressive Sympto*”))	AND	((“Cárie dental” *OR* “Cárie dentária” *OR* “Dental Caries” *OR* “Desmineralização dental” *OR* “Desmineralização dentária” *OR* “Tooth Demineralization” *OR* “Cavidade dental” *OR* “Cavidade dentária” *OR* “Dental Cavit*” *OR* “Dental Decay” *OR* “Tooth Decay”) *OR* (“Doença Periodontal” *OR* “Gengivite” *OR* “Gingivitis” *OR* “Gum Diseas*” *OR* “Periodont*” *OR* “Parodont*”) *OR* (“Perda dental” *OR* “Perda dentária” *OR* “Tooth Loss” *OR* “Edêntulo” *OR* “Toothless” *OR* “Edentul*” *OR* “Dental Status” *OR* “Status Dental”) *OR* (“Saúde bucal” *OR* “Saúde oral” *OR* “Oral Health” *OR* “Dental Health”))
PsycInfo	(“Depression” *OR* “depressive disorder not otherwise specified” *OR* “depressive symptom*” *OR* “Mental Health” *OR* “Mental Hygiene” *OR* “Mental Disord*” *OR* “Common Mental Disord*”)	AND	(“Dental Caries” *OR* “Periodont*” *OR* “Tooth Loss” *OR* “Edentul*” *OR* “Dental Status” *OR* “Oral Health”)
PubMed	((“Mental Health”[MeSH] *OR* “Mental Health”) *OR* (“Mood disorders”[MeSH] *OR* “Mood disord*”) *OR* (“Common Mental Disord*”) *OR* (“Depression”[MeSH] *OR* “Depression” *OR* “Depressive Disorder”[MeSH] *OR* “Depressive Disord*” *OR* “Depressive Sympto*”))	AND	((“Dental Caries”[MeSH] *OR* “Dental Caries”OR “Tooth Demineralization” *OR* “Dental Cavit*” *OR* “Dental Decay” *OR* “Tooth Decay”) *OR* (“Periodontal Diseases”[Mesh] *OR* “Gingivitis” *OR* “Gum Diseas*” *OR* “Periodont*” *OR* “Parodont*”) *OR* (“Tooth Loss”[MeSH] *OR* “Tooth Loss” *OR* “Toothless” *OR* “Edentul*” *OR* “Dental Status”) *OR* (“Oral Health”[MeSH] *OR* “Oral Health” *OR* “Dental Health”))
SciElo	((saúde mental) *OR* (mental health) *OR* (transtornos de humor) *OR* (mood disorders) *OR* (transtornos mentais comuns) *OR* (common mental disorders) *OR* (depressão) *OR* (depression) *OR* (sintomas depressivos) *OR* (sintomas de depressão) *OR* (depressive symptoms) *OR* (transtornos depressivos) *OR* (depressive disorders))	AND	((cárie dentária) *OR* (dental caries) *OR* (desmineralização dentária) *OR* (tooth demineralization) *OR* (cavidades dentárias) *OR* (dental cavities) *OR* (decadência dentária) *OR* (dental decay) *OR* (tooth decay) *OR* (doenças periodontais) *OR* (periodontal diseases) *OR* (gengivite) *OR* (gingivitis) *OR* (doenças gengivais) *OR* (gum diseases) *OR* (periodont*) *OR* (parodont*) *OR* (perda dentária) *OR* (tooth loss) *OR* (edentulismo) *OR* (toothless) *OR* (status dental) *OR* (dental status) *OR* (saúde bucal) *OR* (oral health) *OR* (saúde dental) *OR* (dental health))
Scopus	TITLE-ABS-KEY (“Mental Health” *OR* “Mental Hygiene” *OR* “Mood Disord*” *OR* “Common Mental Disord*” *OR* “Depression” *OR* “Depressive Disord*” *OR* “Depressive Symptom*”)	AND	TITLE-ABS-KEY (“Dental Caries” *OR* “Tooth Demineralization” *OR* “Dental Cavit*” *OR* “Dental Decay” *OR* “Tooth Decay” *OR* “Periodontal Diseas*” *OR* “Gingivitis” *OR* “Gum Diseas*” *OR* “Periodont*” *OR* “Parodont*” *OR* “Tooth Loss” *OR* “Toothless” *OR* “Edentulism” *OR* “Dental Status” *OR* “Oral Health” *OR* “Dental Health”)
Web of Science	(“Depression” *OR* “Depressive Disord*” *OR* “Depressive Symptom*” *OR* “Common Mental Disord*” *OR* “Mental Health” *OR* “Mental Hygiene”)	AND	(“Dental Caries” *OR* “Tooth Demineralization” *OR* “Dental Cavit*” *OR* “Dental Decay” *OR* “Tooth Decay” *OR* “Gingivitis” *OR* “Gum Diseas*” *OR* Periodont* *OR* Parodont* *OR* “Tooth Loss” *OR* “Toothless” *OR* “Edentul*” *OR* “Dental Status” *OR* “Oral Health” *OR* “Dental Health”)

### Study selection

The selection process followed a three-stage screening approach. The Rayyan software was used throughout. Two independent reviewers (MKM and MCR) screened records in parallel, blinded to each other’s decisions. At the end of each stage, disagreements were resolved by consensus.

### Data collection and quality assessment

Data extraction was performed independently by the two reviewers using a standardized extraction form. Extracted information from each included study comprised: author(s), publication year, journal, country of study and sample characteristics, analytical sample size, study design, assessment and operationalization of exposure and outcome, direction of association, statistical analysis performed, confounding factors accounted for, adjusted effect measures of interest and respective confidence intervals, and quality assessment scores.

Quality assessment followed the Joanna Briggs Institute (JBI) Critical Appraisal Checklist^13 14^ appropriate to each design. Each criterion was rated as Yes, No, Unclear, or Not Applicable, with the total score calculated as the percentage of Yes responses over the applicable ones.

### Synthesis methods

Studies were grouped according to two main organizing principles: a) the oral health condition examined, and b) the directionality of the association investigated (oral health condition as exposure or outcome, and the same for symptoms of depression). This bidirectional categorization resulted in six different groups for qualitative and, when possible, quantitative synthesis.

Due to the limited number of studies for some associations or incompatible effect measures for pooling, a meta-analysis was feasible in only four of the six groups. For these, pooled odds ratios (OR) were calculated with a 95% confidence interval using random-effects models to account for heterogeneity, which was assessed through I^2^. Sensitivity analyses were performed to estimate the effect of each study on the pooled results. Subgroup analyses were conducted when appropriate.

Studies presenting adjusted beta coefficients were converted through exponential transformation to odds ratios for comparability. Effect measures that were not compatible or convertible, such as means ratios (MR), were not considered, nor were unadjusted estimates. Furthermore, whenever studies reported data derived from the same samples (for instance, overlapping NHANES cycles), a single study was selected for the meta-analyses to avoid double counting, and their counterparts were included only in the narrative synthesis. Due to the low number of studies per group, meta-regression, funnel plot, and Egger tests were not performed. All analyses were conducted using STATA 15.1 (StataCorp, College Station, USA).

## Results

### Study selection

**Figure 1:**
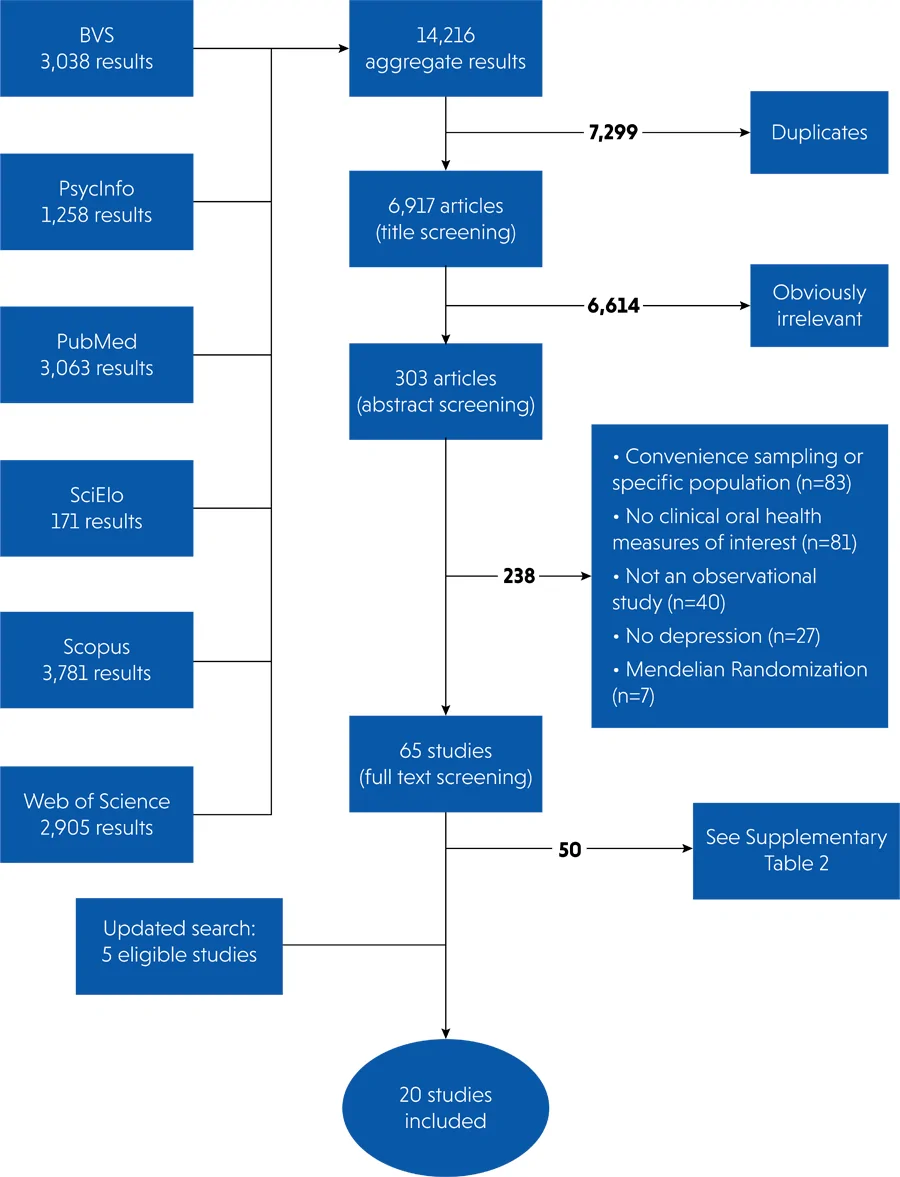
PRISMA flow diagram of the study selection process.

The database search yielded 14,216 records. After removing duplicates (n = 7,299) and screening titles and abstracts, 65 articles remained for full-text appraisal. Of these, 50 were excluded (detailed reasons in Table 2), resulting in 15 studies included in the review. An updated search conducted in February 2026 yielded another 5 studies, for a total of 20. The selection process is illustrated in Figure 1.

**Table 2:** Main reasons for exclusion after full-text appraisal.

Study	Setting	Main reason for exclusion
Abbas et al., 2025^42^	Japan	No measure for depressive symptoms
Abregú-Flores et al., 2025^43^	Spain	Convenience sampling or specific population
Alkan et al., 2013^44^	Turkey	Convenience sampling or specific population
Anttila et al., 2001^45^	Finland	No adjusted effect measure between exp/out of interest
Barbosa et al., 2018^46^	Brazil	Convenience sampling or specific population
Cankovic et al., 2019^47^	Serbia	No clinical OH measure of interest
Castro et al. 2006^48^	Brazil	Convenience sampling or specific population
Castro et al., 2003*	Brazil	Convenience sampling or specific population
Coelho et al., 2019^49^	Brazil	Convenience sampling or specific population
D’Ávila et al., 2017^50^	Brazil	No adjusted effect measure
Fatima et al., 2016^51^	India	Convenience sampling or specific population
Fukuhara et al., 2020^52^	Japan	No adjusted effect measure
Gluszek-Osuch et al., 2024^53^	Poland	Convenience sampling or specific population
Hajek et al., 2023^54^	Europe/Israel	No clinical OH measure of interest
Hao et al., 2024^55^	USA	Insufficient information on sample
Hsu et al., 2015^56^	Taiwan	Medical records
Huang et al., 2024^57^	USA	No adjusted effect measure
Hybels et al., 2015^58^	USA	No clinical OH measure of interest
Jacob et al., 2020^59^	Spain	No clinical OH measure of interest
Katebi et al., 2025^60^	Iran	Insufficient information on MH assessment
Khambaty et al., 2013^61^	USA	No measure for depression symptoms
Kim et al., 2021^62^	South Korea	No clinical OH measure of interest
Kunrath et al., 2020^63^	Brazil	Convenience sampling or specific population
Li et al., 2011^64^	China	Convenience sampling or specific population
Liu et al., 2025^65^	China	No clinical OH measure of interest
Llaneza et al., 2026^66^	USA	No clinical OH measure of interest
Menezes-Silva et al., 2016^67^	Brazil	Convenience sampling or specific population
Moghadam et al., 2015^68^	Iran	Convenience sampling or specific population
Mohammadi et al., 2019	Iran	No adjusted effect measure
Moss et al., 1996	USA	No adjusted effect measure
Nakazawa et al., 2024^69^	Japan	No clinical OH measure of interest
Okoro et al., 2011	Japan	No clinical OH measure of interest
Park et al., 2013^70^	South Korea	No clinical OH measure of interest
Persson et al., 2003^71^	USA	Convenience sampling or specific population
Powell et al., 2024^72^	USA	No clinical OH measure of interest
Roohafza et al., 2015	Iran	Convenience sampling or specific population
Rosalin et al., 2019	Brazil	Convenience sampling or specific population
Saletu et al., 2006^73^	Austria	Insufficient information on sample
Sersen et al., 2025	Chile	No measure for depression symptoms
Shi-Jia et al., 2025^74^	China	No clinical OH measure of interest
Solis et al., 2004 (1)	Brazil	Convenience sampling or specific population
Solis et al., 2004 (2)	Brazil	Full text not found
Sperr et al., 2017	Austria	Convenience sampling or specific population
Sundaranjan et al., 2015	India	Insufficient information on sample
Takiguchi et al., 2015	Japan	Convenience sampling or specific population
Tay et al., 2025	Singapore	No clinical OH measure of interest
Urzua et al., 2012^75^	Chile	No measure for depression symptoms
Vasiliu et al., 2025^76^	Romania	Convenience sampling or specific population
Viana et al., 2013^77^	Brazil	Convenience sampling or specific population
Wei et al., 2024	USA	No clinical OH measure of interest
Wiener et al., 2015^78^	USA	No clinical OH measure of interest
Yang et al., 2015^79^	South Korea	No clinical OH measure of interest
Yang et al., 2016 (1)^80^	South Korea	No clinical OH measure of interest
Yang et al., 2016 (2)^81^	South Korea	No measure for depression symptoms
Zemedikun et al., 2021	UK	Medical records
Zhang et al., 2012	China	No clinical OH measure of interest
Zhang et al., 2025	China	No clinical OH measure of interest

OH: oral health; MH: mental health (depressive symptoms).

Twenty studies met the criteria defined *a priori* and were included in this review. Their publication dates ranged from 2012 to 2026, and although no language constraints were applied, all selected studies were published in English. As some studies addressed more than one oral health condition of interest, there were a total of 26 distinct analyses examining the associations of interest across different directionalities. The vast majority employed a cross-sectional design (n = 16), one was a case-control study,^15^ and three were prospective studies ranging from one year^16^ to 20 years of follow-up.^17^

Geographically, the included studies represented diverse populations across four continents; however, there was a bias toward high-income countries. In particular, the United States contributed the most studies (n = 8), all utilizing data from the National Health and Nutrition Examination Survey (NHANES) across different waves.^18-21^

Sample sizes varied considerably, ranging from 300 participants^15^ to 62,119.^22^ Most studies focused on adult population, with four specifically examining older adults (either 60 or 65 years and older). As per inclusion criteria, all samples were community-dwelling and non-institutionalized.

Regarding directionality, since four studies investigated multiple conditions,^18 23-25^ 21 analyses were extracted, with 11 examining depressive symptoms as the exposure to oral health outcomes, and 10 investigating oral health conditions as exposures to symptoms of depression used as the outcome. The distribution of oral health conditions was balanced among periodontal disease (n = 7), dental caries (n = 7), and tooth loss (n = 7). This balance does not persist when examining associations by directionality.

### Quality assessment

The methodological quality of included studies was assessed using the JBI Critical Appraisal Checklists. Overall, the screened studies demonstrated fair to high quality, with scores ranging from 77.8% to 100% of applicable criteria met.

Of the sixteen cross-sectional studies, eleven yielded a perfect score (8/8). The three cross-sectional studies with less than perfect scores^23 24 26^ all yielded 7/8 (87.5%), with shortcomings in exposure or outcome measurement and reliability, and partially limited or unclear rationale for confounder adjustments.

The three prospective cohort studies showed strong but varied quality scores. Chou et al.^22^ and Chu et al.^17^ both achieved 82% (9/11 criteria), with limitations primarily related to incomplete follow-up documentation and lack of strategies to address missing data. Nascimento et al.^16^ scored 80% (8/10 applicable criteria), with its main limitation being the short one-year follow-up period between depression assessment and periodontal examination.

The single case-control study^15^ met 77.8% of applicable criteria (7/9). The quality score was lower due to a lack of matching between cases and controls and unclear inter-examiner reliability among the nine dentists conducting examinations – an issue that is potentially aggravated by the relatively small sample size (n = 300), meaning fewer than 35 participants per examiner.

An important limitation is the scarcity of prospective studies (3 out of 20). Most studies (n = 18) provided comprehensive adjustment for key confounders, including age, sex, socioeconomic factors, and health behaviors such as smoking, though with some variation across studies. Additionally, heterogeneity in operational definitions, particularly for periodontal disease classification and depression severity cutoff points, posed challenges for direct comparisons across studies.

Finally, there is an important caveat: while useful for systematic and replicable evaluation, any quality assessment instrument has inherent limitations in reflecting certain methodological nuances. For instance, Skoskiewicz-Malinowska et al.^25^ achieved a perfect 8/8 score, despite not reporting the thresholds used for Clinical Attachment Loss categories or the sample size for the analysis involving periodontal disease.

### Synthesis of results by association

Tables 3 and 4 summarize the extracted information from each of the 20 studies. The subsections below explore their analyses by investigated association.

**Table 3:** Main characteristics of selected studies with oral health as outcome.

Author/year	Country/study design	Sample characteristic	Measure of depression (cutoff)	Measure of oral health (cutoff)	Statistical analysis (effect measure)	Adjustment variables	Results	JBI score (%)
Periodontal disease								
Aldosari et al., 2020^18^	USA/ Cross-sectional	9799 adults ≥ 30 years	PHQ-9 divided into 4 categories	CDC/AAP criteria based on probing depth (PD) and clinical attachment loss (CAL) measurements at interproximal sites; any periodontitis and 3 categories of severity	Logistic regression; Odds Ratio (OR)	Age, sex, race/ethnicity, education, income, smoking, alcohol consumption, psychotherapeutic medications, and diabetes.	Severe depressive symptoms associated w/ mild periodontitis (AOR = 2.20, 99%CI: 1.03–4.66). No other significant associations.	100
		NHANES 2009-14						
Al-Joghaiman et al., 2025^28^	USA/ Cross-sectional	2764 adults ≥ 18 years	Single question from NHANES questionnaire, 15-day time window (4 categories)	CDC/AAP criteria based on PD and CAL; any periodontitis and 3 categories of severity	Logistic regression; Odds Ratio (OR)	Age, sex, race/ethnicity, education, income, BMI, alcohol consumption, diabetes, last dental consultation and dental insurance	Depressive symptoms were not significantly associated to periodontal disease. Results for category 2 of severity were higher than for 1 or 3 (AOR = 1.17, 35%CI: 0.53–2.47)	75
		NHANES 2013-14						
Delgado-Angulo et al., 2015^23^	Finland/ Cross-sectional	4673 adults ≥30 years	BDI score divided into 3 categories	Number of teeth w/ periodontal any periodontal pocket ≥4mm	Negative binomial regression; Rate Ratios (RR)	Age, sex, marital status, education, income, diabetes, coronary heart disease, oral health behaviors (sugar consumption, toothbrushing frequency, dental attendance, smoking), plaque accumulation, psychotropic medication use.	RR = 1.25 (95%CI 1.07–1.45)	87.5
		Participants of the Health 2000 Survey						
Hwang & Park, 2018^26^	South Korea/Cross-sectional	4328 adults ≥ 30 years		Any periodontal disease (CPI score > 1) and severe periodontal disease (CPI > 3)	Hierarchical Poisson regression; Prevalence Ratios (PR)	Age, gender, schooling, income, location, marital status, oral hygiene, medication intake, smoking, dental plaque, saliva flow, number of teeth	No significant associations, except in the age stratified analysis (20–29 years)	87.5
		KNHANES VI						
Jungo et al., 2025^27^	USA/ Cross-sectional	9537 adults ≥ 30 years	PHQ-9 dichotomized at ≥ 10	CDC/AAP criteria based on PD and CAL; any periodontitis and 3 categories of severity	Logistic regression; Odds Ratio (OR)	Age, gender, schooling, income, tobacco use, chronic health conditions	Model 1 (unadjusted for SES): OR = 1.26 (95%CI: 1.01–1.56)	100
		NHANES					Model 7 (Adjusted for SES): OR = 0.94 (95%CI: 0.75–1.18)	
		2009-14						
Nascimento et al., 2018^16^	Brazil/Prospective	539 adults 31 years-old	BDI-II divided into 3 categories and dichotomized at ≥14	CDC/AAP criteria dichotomized as any periodontal condition (mild, mod, severe) and as ≥moderate	Parametric g-formula (total, natural direct/indirect, and controlled direct effects);	Sex, maternal schooling at birth, BMI and individual schooling at age 30, smoking status.	Direct effect RR = 1.18 (95%CI 1.03–1.36);	90
		1982 Pelotas Cohort			Risk Ratios (RR)	Mediators: C-reactive protein levels and dental flossing frequency - neither mediated the associations	Controlled direct effect RR = 1.12 (95%CI 1.00–1.28)	
Dental caries								
Aldosari et al., 2020^18^	USA/ Cross-sectional	7011 adults ≥ 30 years	PHQ-9 divided into 4 categories	Number of teeth w/ untreated lesions (continuous)	Logistic regression; Odds Ratio (OR)	Age, sex, race/ethnicity, education, income, smoking, alcohol consumption, psychotherapeutic medications, and diabetes	No significant association.	100
		NHANES 2009-14		Presence (binary)	Poisson regression; Means Ratio (MR)			
Delgado-Angulo et al., 2015^23^	Finland/ Cross-sectional	4666 adults ≥ 30 years	BDI score	Number of decayed teeth (continuous)	Negative binomial regression; Rate Ratios (RR)	Age, sex, marital status, education, income, diabetes, coronary heart disease, oral health behaviors (sugar consumption, toothbrushing frequency, dental attendance, smoking), plaque accumulation, psychotropic medication use.	RR 0.96 (95%CI: 0.88–1.05)	87.5
		Participants of the Health 2000 Survey	Divided into 3 categories					
Hugo et al., 2012^32^	Brazil/ Cross-sectional	390 older adults (≥ 60 years)	GDS-15 dichotomized at ≥6.	D component of DMFT dichotomized at ≥1	Hierarchical Poisson regression; Prevalence Ratios (PR)	Age, gender, schooling, income, location, marital status, oral hygiene, medication intake, smoking, dental plaque, saliva flow, number of teeth	PR = 1.31 (95%CI: 1.07–1.60)	100
Wiener et al., 2018^20^	USA/ Cross-sectional	3217 adults from 21 to 64 years	PHQ-9 divided into 3 categories and dichotomized at ≥10	Presence of any coronal caries (binary)	Logistic regression; Odds Ratio (OR)	Age, sex, race, education, income, health insurance, dental visit, history of previous restorations	Mild depression: OR = 1.27 (95% CI: 0.96, 1.69)	100
		NHANES 2013-14					Moderate/severe depression: OR = 1.61 (95%CI: 0.95–2.73)	
Xie et al., 2024^21^	USA/ Cross-sectional	8740 adults ≥ 20 years	PHQ-9 divided into 3 categories and dichotomized at ≥ 10	Presence of any dental caries (binary)	Logistic regression; Odds Ratio (OR)	Age, sex, race, education, income, diabetes, smoking, dental visits, number of teeth, C-reactive protein, sugar intake, filled caries status	Mild depression: OR 1.07 (95%CI: 0.85–1.34)	100
		NHANES 2015-18					Moderate/severe depression: OR 1.06 (95%CI: 0.83–1.36)	
Tooth loss								
Aldosari et al., 2020^18^	USA/ Cross-sectional	7011 adults ≥ 30 years	PHQ-9 divided into 4 categories	Number of lost teeth (continuous)	Poisson regression; Means Ratio (MR)	Age, sex, race/ethnicity, education, income, smoking, alcohol consumption, psychotherapeutic medications, and diabetes.	Mild (MR = 1.20, 99% CI: 1.06-1.37) and moderate (MR = 1.38, 99% CI: 1.15-1.66) depressive symptoms associated w/ higher mean number of missing teeth.	100
		NHANES 2009-14						
	
Al-Zahrani et al., 2020^19^	USA/Cross-sectional	22,532 adults ≥ 18 years	PHQ-9 dichotomized at ≥10	Divided into 3 categories of functional dentition (5 categories in supplementary analysis	Multinomial logistic regression; Odds Ratio (OR)	Age, gender, race, socioeconomic status, education, BMI, diabetes, smoking, alcohol intake	All results in main and supplementary analysis were significant.	100

**Table 4:** Main characteristics of selected studies with oral health as exposure.

Author/year	Country/study design	Sample characteristic	Measure of depression (cutoff)	Measure of oral health (cutoff)	Statistical analysis (effect measure)	Adjustment variables	Results	JBI score
Periodontal disease								
Hozuri et al., 2020^15^	Iran/ Case-control	300 older adults ≥ 60 yearw	GDS-15 dichotomized at ≥5 and as a continuous variable	Ramfjord teeth examined	Logistic regression; Odds Ratio (OR)	Age, gender, education level, smoking	OR = 1.29 (95% CI: 0.74-2.24)	77.8%
		100 cases, 200 controls		PDI ≥4				
Dibello et al., 2025^30^	USA/ Cross-sectional	551 older adults ≥ 60 years	Two questions from NHANES questionnaire, 15-day time window (4 categories), adapted from PHQ-2	CDC/AAP criteria based on PD and CAL; any periodontitis and 3 categories of severity	Logistic regression; Odds Ratio (OR)	Age, gender, education, income, tobacco and alcohol use, chronic health conditions	Moderate periodontitis: OR = 0.90 (95%CI: 0.56–1.46)	100%
		NHANES 2009-14					Severe: OR = 1.38 (95%CI: 0.69– 2.76)	
Skoskiewicz-Malinowska et al., 2018^25^	Poland/ Cross-sectional	Unreported sample size; subjects with ≥2 natural teeth	PHQ-9 divided into 5 categories	% of sites with bleeding; ≥4mm pocket depth; and CAL (threshold not specified)	Linear regression; Beta (β)	Age, gender, number of diseases, medications, living conditions, education, income	No significant results. Did not present adjusted effect measures.	100%
Walther et al., 2023^31^	Germany/Cross-sectional	5591 participants of the Hamburg City Health Study, between 45–75 years	PHQ-9 as a continuous score and divided into 5 categories	3 severity levels of periodontitis according to Eke et al. (2012) criteria	Linear regression with log transformed depressive score; Beta (β)	Age, sex, diabetes, education, smoking, antidepressant medication	Severe periodontitis: β = 0.72 (95%CI: 0.21–1.23)	100%
							Severe periodontitis×age: β = -0.01 (95%CI: -0.02 to -0.00)	
Dental caries								
Palomer et al., 2024^24^	Chile/ Cross-sectional	2953 adults ≥18 years from the Chilean Health Survey 2016-17	CIDI-DSM criteria	Number of decayed or filled teeth (continuous)	Logistic regression; Odds Ratio (OR)	Age, sex and educational level	No significant association: OR 1.06 (95%CI: 0.97–1.16)	87.5%
Skoskiewicz-Malinowska et al., 2018^25^	Poland/ Cross-sectional	500 older adults ≥ 65, Wroclaw	PHQ-9 divided into 5 categories	Decayed + Missing + Filled teeth (continuous)	Linear regression; Beta (β)	Age, gender, number of diseases, medications, living conditions, education, income	β = 0.11 (95%CI: 0.02 to 0.20)	100%
Tooth loss								
Chou et al., 2025^22^	Taiwan/Prospective	62,119 older adults (≥65) participants of annual physical examination program in Taipei City	BSRS-5 dichotomized at ≥6	4 categories of functional dentition (≥20 teeth; 10-19; 1-9; edentulism);	Generalized Estimating Equations; Odds Ratio (OR)	Age, sex, education, income, marital status, BMI, comorbidities, lifestyle and baseline BSRS-5 score	Number of teeth at baseline (vs ≥20 teeth): 10–19: OR 1.09 (95%CI: 1.00–1.18)	81.8%
		9-year follow-up		Changes in number of teeth between follow-ups (no loss; 1 to 4; 5 to 9; and ≥10)			1–9: OR 1.11 (95%CI: 1.01–1.23)	
							No teeth: OR 1.23 (95%CI: 1.12–1.34)	
							Changes in number of teeth:	
							1–4: OR 1.42 (95%CI: 1.34–1.51)	
							5–9: OR 1.55 (95%CI: 1.41–1.69)	
							≥ 10: OR 1.70 (95%CI: 1.55–1.85)	
Chu et al., 2023^17^	Japan, Prospective	1668 adults ≥ 40 years	CES-D scale (0-60) dichotomized at ≥16	3 categories of functional dentition (≥20 teeth; 10–19; and 0 to 9 teeth)	Generalized Estimating Equations; Odds Ratio (OR)	Age, sex, education, marital status, satisfaction w/ economic situation, smoking, alcohol intake, leisure time activity, denture use, self-rated health, activities of daily living, hypertension, heart disease, diabetes, and baseline CES-D	10–19: OR: 1.42 (95%CI: 0.87–2.31)	81.8%
		20-year follow-up					< 10: OR: 1.60 (95%CI: 1.03–2.51)	
Dibello et al., 2025^34^	USA/ Cross-sectional	551 older adults ≥60 years	Two questions from NHANES questionnaire, 15-day time window (4 categories), adapted from PHQ-2	20 or more (reference); 10–19; 1–9 and edentulism.	Logistic regression; Odds Ratio (OR)	Age, gender, education, income, tobacco and alcohol use, chronic health conditions	10–19: OR: 0.77 (95%CI: 0.41–1.43)	100%
		NHANES 2009-14					1–9: OR: 1.10 (95%CI: 0.46–2.66)	
							Edentulism: OR: 1.26 (95%CI: 0.67–2.36)	
Koh et al., 2025^41^	South Korea/Cross-sectional	9166 adults (cutoff age unreported)	Single question derived from the CIDI-SF, dichotomized	Dichotomized at <12 and ≥12 remaining teeth	Logistic regression; Odds Ratio (OR)	Age, gender, education, income, tobacco use, hypertension and hypertriglyceridemia, need of prosthetic rehabilitation	OR = 1.45 (95%CI: 1.12–1.87)	87.5%
		KNHANES VI 2013-15						
Ortuño et al., 2024^33^	Chile/ Cross-sectional	5383 participants ≥15 years from the Chilean Health Survey 2016-17	CIDI-DSM criteria	Remaining teeth dichotomized at < 20; Anterior tooth loss (binary)	Logistic regression with mediation analysis; Odds Ratio (OR)	Age, sex, education, smoking, diabetes	No significant associations	100%
Palomer et al., 2024^24^	Chile/ Cross-sectional	2953 adults ≥18 years from the Chilean Health Survey 2016-17	CIDI-DSM criteria	Number of teeth remaining (continuous); Anterior loss (binary)	Logistic regression; Odds Ratio (OR)	Age, sex and educational level	No significant associations	87.5%
Skoskiewicz-Malinowska et al., 2018^25^	Poland/ Cross-sectional	500 older adults ≥ 65, Wroclaw	PHQ-9 divided into 5 categories	Number of missing teeth (continuous)	Linear regression; Beta (β)	Age, gender, number of diseases, medications, living conditions, education, income	β = 0.111 (95%CI: 0.105–0.116)	100%

#### Depression → periodontal disease

Six studies evaluated the association of depressive symptoms as exposure and periodontal disease as outcome,^16 18 23 26^ with sample sizes ranging from 539 to 9,799.

The assessment of depression symptoms varied across studies. Two studies used the PHQ-9,^18 26^ but with different operationalizations. Aldosari et al.^18^ and Jungo et al.^27^ adopted 4 categories, while Hwang and Park^26^ dichotomized the score, using a cut-off of 10 or more. Al-Joghaiman et al.^28^ used a single question adapted from the PHQ-2. The other two other used different versions of the BDI. The periodontal assessment methods differed even more: Aldosari and Nascimento used comprehensive CDC/AAP criteria, Hwang and Park^26^ used only the Community Periodontal Index, and Delgado-Angulo measured only pocket depth.

The findings were inconsistent. Nascimento et al.,^16^ the only prospective study in this section, was also the only one to find persistent associations after adjustment, reporting significant effects of depressive symptoms on any periodontitis (RR = 1.19, 95%CI: 1.04–1.36) and moderate/severe periodontitis (RR = 1.18, 95%CI: 1.03–1.35).

Among cross-sectional studies, results were largely null. Aldosari et al.^18^ found that the only significant associations were between severe depression and two categories of periodontitis: mild and severe. Al-Joghaiman et al.^28^ reported an OR of 1.17 (95%CI: 0.53 – 2.47). Jungo et al., who, like Aldosari et al.^18^ and Al-Joghaiman et al.^28^ used data from the 2009–2014 cycles of NHANES, also reported a null effect after full adjustment for socioeconomic stats (OR = 0.94, 95%CI: 0.75 – 1.18). Hwang and Park^26^ reported findings that were also non-significant, with the exception of one age group (20–29). Delgado-Angulo et al.^23^ reported a marginal association that disappeared after adjustment (RR = 0.96, 95%CI: 0.88 – 1.05).

**Figure 2:**
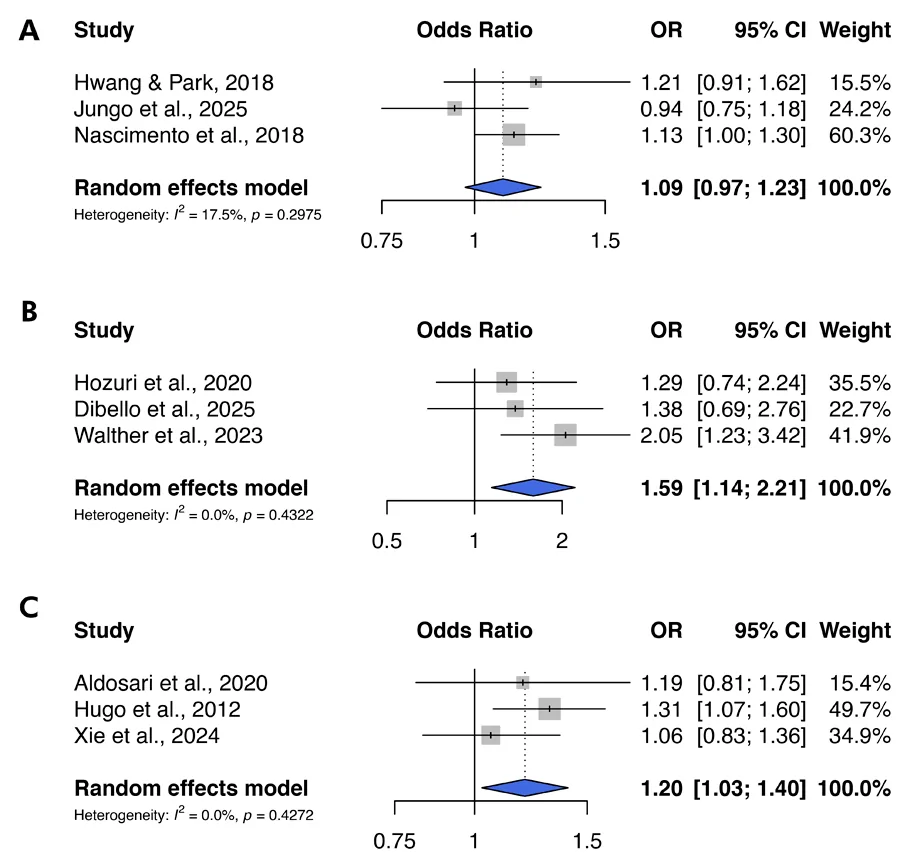
Forest plots of pooled estimates for: periodontal disease as outcome (A), periodontal disease as exposure (B), and dental caries as outcome (C).

**Figure 3:**
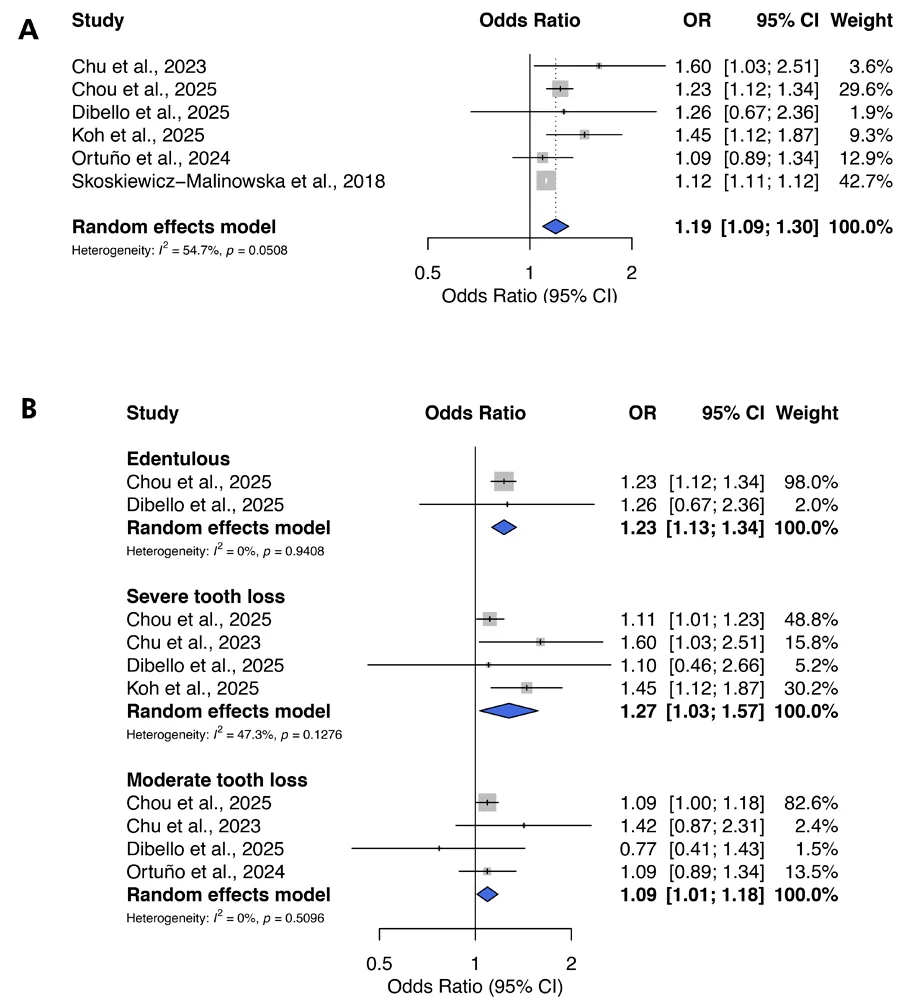
Subgroup analyses of tooth loss as an exposure by age group (A) and study design (B).

For the quantitative analyses, only three studies were pooled. Due to overlapping NHANES samples, only Jungo et al. was selected, as it was the most recent study with the largest sample size. Delgado-Angulo et al. was excluded for presenting risk ratios and means of continuous variables, which precluded comparison with the OR measures. The pooled estimates of the included studies yielded an OR = 1.09 (95%CI: 0.96–1.23, I^2^ = 17.5%), confirming the overall weak and inconsistent evidence for depression leading to periodontal disease (see Figure 2a). Sensitivity analysis showed that removing Jungo et al. would make the association significant by a very slight margin (OR = 1.14, 95%CI: 1.01–1.28). The corresponding graph can be found in Figure 3a.

#### Periodontal disease → depression

Four studies evaluated periodontal disease as an exposure and depressive symptoms as the outcome,^15 25 29^ with sample sizes ranging from 300 to 5,591. Skoskiewicz-Malinowska et al.^25^ did not present the sample size for this analysis. However, since this study adequately presents it in two other analyses, it was included in this synthesis as well.

The PHQ-9 was used by Skoskiewicz-Malinowska et al.^25^ as a continuous score (0–27) and by Walther as a log-transformed score, while Hozuri et al.^15^ used the GDS-15 (specific for the older population) dichotomized at 5 points or more. Dibello et al.^30^ used an adaptation of the PHQ-2. Periodontal assessment methods varied considerably across the four studies: Hozuri et al.^15^ used the dichotomized Periodontal Disease Index (PDI ≥4) to characterize periodontitis, examining only the Ramfjord teeth. Walther et al.^29^ conducted full-mouth examinations using the Eke et al. (2012)^31^ criteria. Skoskiewicz-Malinowska et al.^25^ reported multiple clinical parameters but did not establish clear diagnostic criteria. Dibello et al.^30^ used clinical attachment loss and probing depth to categorize periodontitis into 3 severity categories using CDC/AAP criteria.

The findings were mixed. Hozuri et al.,^15^ the only case-control study included in this review, observed no significant difference, with an adjusted OR of 1.29 (95%CI: 0.74–2.24). Skoskiewicz-Malinowska et al.^25^ also reported null associations between periodontal measurements and depression scores (all regression coefficients encompass zero), though it cannot be overstated that interpretation of these results is limited by the restricted information on exposure thresholds and sample size.

In contrast, Walther et al.^29^ reported a significant association between severe periodontitis and severity of depressive symptoms (β = 0.72, 95%CI: 0.21–1.23).

For the quantitative analysis, Skoskiewicz-Malinowska et al. was excluded, as their effect measures were unadjusted. The β estimates presented by Walther et al. were converted to ORs through exponentiation for comparability. The pooled estimates suggest a significant association (OR = 1.59, 95%CI: 1.14–2.21, I^2^ = 0.0%) (see Figure 2b). However, sensitivity analysis shows that removing Walther et al. would nullify the association (OR = 1.32, 95% CI: 0.85–2.04) (see Figure 3b).

#### Depression → dental caries

Five studies evaluated the relationship between depressive symptoms as the exposure and dental caries as the outcome, with sample sizes ranging from 390 to 8,740 participants. Three^18 20 21^ studies were secondary analyses of different NHANES waves; Delgado-Angulo et al.^23^ conducted a secondary analysis using data from the Health 2000 Survey in Finland. The remaining study was original research conducted in southern Brazil.^32^

Depression assessment was slightly less heterogeneous in this group, with the PHQ-9 used in 3 of the 5 studies (though with different cut-off points). The other studies used validated regional versions of the GDS-15^32^ and the BDI-21^23^ for the Brazilian and Finnish populations, respectively. Aldosari et al.^18^ and Delgado-Angulo et al.^23^ measured the number of teeth with untreated coronal caries (excluding third molars), while Wiener et al.^20^ dichotomized the variable, considering any teeth with untreated coronal caries as a positive outcome. Hugo et al.^32^ used the same dichotomization but focused exclusively on root caries. Xie et al.^21^ uniquely studied both coronal and root dental caries, dichotomizing their presence as did Wiener et al. and Hugo et al.

Hugo et al.^32^ examining older Brazilian adults, reported an adjusted PR of 1.31 (95%CI: 1.07–1.60) for the association between depressive symptoms and radicular caries. Xie et al. also reported a positive association in their root caries analysis, with an OR of 1.25 (95% CI: 1.09–1.66). These results are similar to those reported by Delgado-Angulo et al.^23^ (RR = 1.25, 95%CI: 1.07–1.45), which studied coronal, not root, caries. However, the coronal caries analysis by Xie et al.^21^ showed no significant associations. Aldosari et al.^18^ reported a significant association only for moderate depression and caries presence (OR = 1.61, 95%CI: 1.03–2.52). Wiener et al.^20^ initially found significant associations for mild and moderate/severe depressive symptoms, but these lost significance after adjusting for tobacco use. Although the authors suggest this indicates behavioral mediation, the cross-sectional design means it is unclear whether this behavioral aspect is a potential mediator or precedes the exposure.

For the quantitative analysis, Delgado-Angulo et al. was excluded for only reporting rate ratios based on continuous measurements. Aldosari et al. and Wiener et al. both used NHANES data from overlapping cycles (2009–2014 and 2013–2014, respectively). Therefore, only Aldosari et al. was included, due to its three times larger sample size. The pooled estimates of the remaining three studies were OR = 1.20 (95%CI: 1.04–1.39, I^2^ = 0.0%) (see Figure 2c). However, sensitivity analyses showed that removing either Aldosari et al.^18^ or Hugo et al.^32^ would be enough to nullify the association (see Figure 3c).

Overall, the evidence suggests a mild positive association between depressive symptoms and dental caries, possibly modified by age, caries site, and behavioral characteristics such as tobacco use.

#### Dental caries → depression

Only two studies evaluated dental caries as the exposure and depressive symptoms as the outcome,^24 25^ with samples of 500 older adults and 2,953 adults over 18, respectively.

Both studies used different approaches to assess depression. Palomer et al.^24^ used the CIDI-SF, while Skoskiewicz-Malinowska et al.^25^ used the PHQ-9 as a continuous score. As for caries assessment, Palomer et al.^24^ counted the number of teeth with cavities, defining a cavity as any surface with discontinuity and including filled teeth. Skoskiewicz-Malinowska et al.^25^ considered the D, F, and M components, so missing teeth comprised the variable (which is curious as they ran analyses for tooth loss using the M component but did not analyze decayed or filled teeth).

The findings were mixed. Palomer et al.^24^ found no significant associations, while Skoskiewicz-Malinowska et al.^25^ reported a positive association between DMFT scores and depression (β = 0.11, 95%CI: 0.02–0.20, p=0.01). However, including missing teeth in this analysis likely introduces bias. Furthermore, the presented estimates were not adjusted.

Due to the different effect measures used in the two studies, a quantitative synthesis was not feasible. The evidence for dental caries as an independent risk factor for depression remains insufficient for drawing conclusions.

#### Depression → tooth loss

Only two studies evaluated depressive symptoms as the exposure and tooth loss as outcome,^18 19^ both secondary analyses using NHANES data from different waves, with sample sizes of 7,011 and 22,532, respectively.

Both studies used the PHQ-9 to assess depressive symptoms but with different approaches. Aldosari et al.^18^ employed four categories of severity, while Al-Zahrani et al.^19^ adopted a cut-off point of 10 or more for dichotomization. Aldosari et al. analyzed tooth loss as a continuous variable, whereas Al-Zahrani divided it into categories of functional dentition.^19^

The findings showed remarkably consistent positive associations. According to Al-Zahrani et al.,^19^ depressive symptoms increased the odds of edentulism by 48% (OR = 1.48, 95%CI: 1.16–1.89) and of lacking functional dentition by 43% (OR = 1.43, 95%CI: 1.18–1.75). Similarly, Aldosari et al.^18^ observed a dose-response relationship, with mean ratios of lost teeth increasing with depressive symptom severity: mild (MR = 1.20, 95%CI: 1.09–1.32), moderate (MR = 1.38, 95%CI: 1.20–1.59), and severe (MR = 1.38, 95%CI: 1.04–1.83).

Both studies point in the same direction, indicating depression as a risk factor for tooth loss. However, due to the different effect measures used and insufficient information to estimate an OR from Aldosari et al., a quantitative synthesis was not feasible here.

#### Tooth loss → depression

Seven studies evaluated tooth loss as the exposure and depressive symptoms as the outcome,^17 22 24 25 33 34 41^ with sample sizes ranging from 500 to 62,119 participants. This direction included two of the three prospective studies in the review.

Depression assessment varied widely: Chu et al.^17^ used CES-D; Chou et al.^22^ used BSRS-5 (dichotomized at ≥ 10 and ≥ 6, respectively); Ortuño et al.^33^ and Palomer et al.^24^ both used CIDI-SF (dichotomizing at ≥ 20 and adopting CIDI-DSM criteria for outcome definition, respectively); Skoskiewicz-Malinowska et al.^25^ used PHQ-9 continuously. Dibello et al.^34^ measured depressive symptoms similarly to their twin paper on periodontal disease, using an adapted PHQ-2. Koh et al.^41^ used a single question derived from the CIDI-SF. Most studies categorized the number of remaining teeth by functional dentition, using 20 or more teeth as the reference. Palomer et al.^24^ performed additional analysis considering anterior tooth loss. Koh et al. dichotomized the exposure as having fewer than 12 remaining teeth.

The prospective studies provided the strongest evidence. Chu et al.,^17^ with 20-year follow-up in Japan, found that having fewer than 10 teeth increased depression risk (OR = 1.60, 95%CI: 1.03–2.51) compared to having 20 or more teeth. Chou et al.,^22^ following 62,119 Taiwanese older adults, reported dose-response relationships: being edentulous (OR = 1.23, 95%CI: 1.12–1.34) and progressive tooth loss during follow-up (OR point estimates ranging from 1.42 to 1.70 depending on the number of lost teeth) increased psychological distress risk.

Cross-sectional findings were mixed. Ortuño et al.^33^ did not report significant associations for CIDI-SF suspected depression. Palomer et al.^24^ found no significant associations with tooth count, only a protective effect of denture use. Skoskiewicz-Malinowska et al.^25^ reported a positive association between missing teeth and depression scores (β = 0.111, 95%CI: 0.105–0.116). Koh et al.^41^ reported a significant association (OR = 1.45, 95%CI: 1.12–1.87), while Dibello et al.^34^ found no significant association with any category of tooth loss severity.

**Figure 4:**
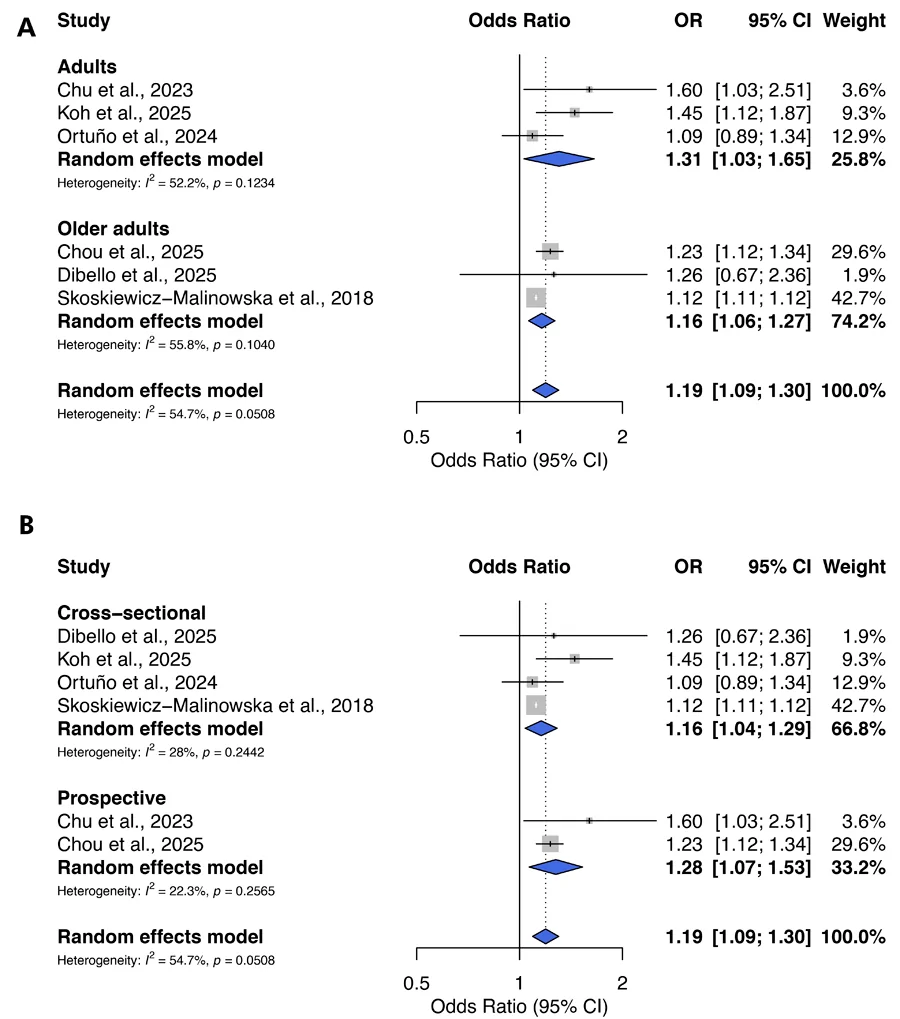
Forest plots of pooled estimates for tooth loss as exposure (A) and subgroup analysis by severity of tooth loss (B).

Due to overlapping samples between Ortuño et al.^33^ and Palomer et al.^24^ (the Chilean National Health Survey 2016–17), Palomer et al. was excluded, as it had half the sample size of Ortuño et al. Therefore, the primary meta-analysis for the association between tooth loss and depressive symptoms included six studies. The pooled estimates indicated a significant association with moderate heterogeneity (OR = 1.19, 95%CI: 1.09–1.30, I^2^ = 54.7%) (see Figure 4a). Sensitivity analyses showed that omitting any study would not nullify the association (see Figure 3d). Notably, when Skoskiewicz-Malinowska et al.^25^ was removed, statistical heterogeneity was almost entirely resolved (I^2^ = 5.8%) and the pooled effect was slightly stronger (OR = 1.24, 95%CI: 1.14–1.35).

Given that many studies in this group used similar categories of tooth loss severity, a subgroup analysis by those categories was conducted. As shown in Figure 3b, there is a clear dose-response gradient: the pooled OR increases with the number of lost teeth, with a narrower CI and higher point estimate among edentulous patients (OR = 1.23, 95%CI: 1.13–1.34, I^2^ = 0.0%, between-group p = 0.204).

**Figure 5:**
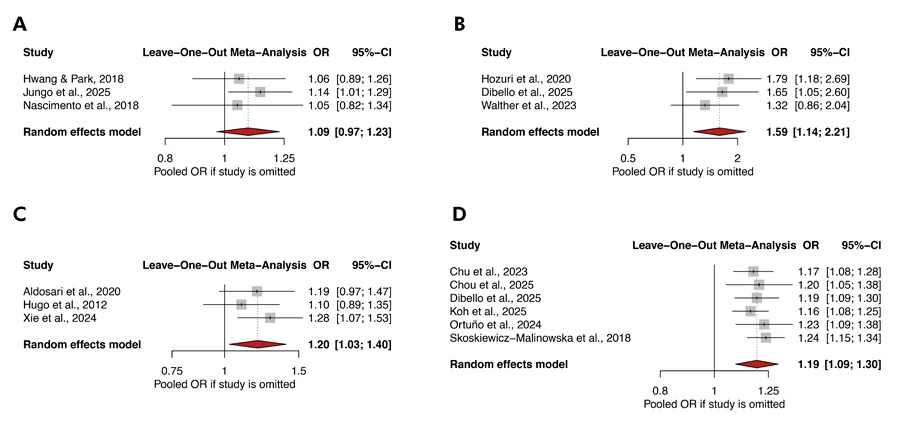
Leave-one-out sensitivity analyses for: periodontal disease as outcome (A), periodontal disease as exposure (B), dental caries as outcome (C), and tooth loss as exposure (D).

Subgroup analyses were also conducted to explore possible differences by study design and age group. Figure 5a shows that the effect magnitude was significant for both adults (pooled OR = 1.31; 95% CI:1.03–1.65) and older adults (OR = 1.16; 95% CI: 1.07–1.25), with no significant heterogeneity between subgroups (p = 0.346). Furthermore, subgroup analysis by study design (Figure 5b) demonstrated that the significant positive association persisted regardless of whether estimates were derived from cross-sectional surveys (OR = 1.15; 95%CI: 1.04–1.27) or prospective cohorts (OR = 1.28; 95%CI: 1.07–1.53; between-group p = 0.323).

## Overall synthesis

Across the six directional associations examined, the integration of quantitative and narrative synthesis reveals a complex interplay, yet emerging patterns are evident. The strongest and most robust associations were observed when oral health conditions were exposures to depressive symptoms. Specifically, our meta-analyses revealed a highly consistent association between severe tooth loss and increased odds of depression (pooled OR = 1.24), supported by a dose-response gradient and corroborated by prospective longitudinal cohorts. Furthermore, our quantitative synthesis demonstrated a significant positive association between severe periodontal disease and depressive symptoms (pooled OR = 1.59).

Regarding depression as an exposure, the evidence appears to be condition-specific: the meta-analysis confirmed that depressive symptoms significantly increase the odds of presenting with untreated dental caries (pooled OR = 1.20), but pooled estimates for depression leading to periodontal disease were not significant. As for depression as a risk factor for tooth loss, although a quantitative synthesis was not feasible, the narrative review indicates an existing relationship, which is intuitive as caries are a main risk factor for tooth loss and may be an important mediator of this association.

Finally, heterogeneity in measurement approaches, especially for periodontal disease, and the use of different thresholds for defining the presence and severity of depressive symptoms across studies, combined with the predominance of cross-sectional designs, limit definitive conclusions on directionality. More than simply calling for further prospective studies, we recommend that authors present supplementary analyses using different cut-off points for commonly used psychometric instruments to improve comparability, enable future meta-analyses with greater accuracy, and further elucidate the temporal dynamics of these possibly bidirectional relationships.

## Discussion

Both depression and poor oral health outcomes are public health problems that increasingly strain public health systems. The evidence reveals a complex relationship between these health domains, varying by oral condition and direction of association, with important implications for understanding each process individually and in combination. The interplay between depression and oral health is likely mediated by complex behavioral and biological pathways. Biologically, the “inflammation hypothesis” suggests that pro-inflammatory cytokines (e.g., IL-1β, IL-6, TNF-α) upregulated in periodontal disease may cross the blood-brain barrier, potentially influencing the pathophysiology of depression^35^. Conversely, depression is associated with dysregulation of the hypothalamic-pituitary-adrenal (HPA) axis and immune function, potentially increasing susceptibility to oral infections.^36^ However, behavioral confounders play a massive role. Socioeconomic disadvantage and poor health literacy are common causes for both conditions.^7^ Depressive symptoms often lead to neglected oral hygiene, cariogenic diet, and smoking, which directly cause oral disease.^37^ These shared risk factors make it challenging to disentangle direct causality from residual confounding in observational studies.

When exploring depression as a risk factor for oral diseases, our meta-analytical findings strongly support the behavioral pathway hypothesis, particularly regarding dental caries. Individuals with depression are thought to have altered mood and be less attentive to self-care. Since oral diseases such as dental caries and tooth loss are related to lack of self-care, depression would be a risk factor for both. Indeed, our quantitative synthesis confirmed a statistically significant positive association between depressive symptoms and untreated dental caries (pooled OR = 1.20). Furthermore, the narrative synthesis strongly supports depression as a predictor for tooth loss, exhibiting a clear dose-response gradient according to depression severity. Conversely, the pooled estimate for depression leading to periodontal disease did not reach statistical significance. Despite this, the behavioral pathway hypothesis remains valid, as the stronger association with tooth loss may reflect the cumulative effect over time, with tooth loss as the ultimate outcome, for which dental caries and periodontal disease are the most likely mediators. It is notable that the only study to report a significant positive association of depressive symptoms as a risk factor was a prospective study.

Regarding the specific association with tooth loss, which showed the strongest consistency in our results, distinct mechanisms are involved. Tooth loss, especially severe tooth loss and edentulism, reduces masticatory function, which influences dietary choices and nutritional intake^38^. It may also negatively affect self-esteem, particularly with anterior tooth loss. Our meta-analysis provided robust evidence supporting this association, demonstrating a 24% increase in the odds of depressive symptoms among individuals with severe tooth loss (Pooled OR = 1.24). Importantly, our subgroup analyses reinforced a dose-response gradient and confirmed that this effect persists regardless of age group or study design. Interestingly, Chou et al.^22^ reported a protective effect of denture wearing, supporting the functional and psychosocial role of dental rehabilitation in preventing depressive symptoms.

An important limitation of this review is the scarcity of prospective studies (only 3 out of 20). Most studies (n = 18) provided comprehensive adjustment for key confounders, including age, sex, socioeconomic factors, and health behaviors such as smoking, though with some variation across studies. Additionally, heterogeneity in operational definitions, particularly for periodontal disease classification and depression severity cutoff points, posed challenges for direct comparisons across studies. We assessed the overall certainty of the evidence using the GRADE approach. The certainty of evidence for the bidirectional associations is considered low to very low. This classification is primarily due to the predominance of cross-sectional designs (16 out of 20 studies), which limits causal inference (imprecision and risk of bias), and substantial heterogeneity in outcome measurement (inconsistency). While prospective studies on tooth loss provide moderate certainty, findings regarding periodontal disease and caries are heavily constrained by inconsistent definitions and lack of temporal data.

However, a major methodological strength of our review is the robust analytical approach used to address this heterogeneity. The inclusion of mathematically converted continuous estimates (Beta coefficients) into odds ratios allowed for a more comprehensive synthesis of the available evidence but inevitably introduced statistical variance. To address this, random-effects leave-one-out sensitivity analyses were performed. These analyses demonstrated that the significant associations were not artifacts of data transformation, as the removal of converted studies entirely resolved statistical heterogeneity without altering the clinical direction of the pooled effects, even though the association sometimes lost significance. Finally, while publication bias is a recognized threat to systematic reviews, formal evaluation through visual inspection of Funnel Plots or statistical tests of asymmetry was not performed. This decision followed the Cochrane Handbook recommendations,^39^ which state that such tests lack sufficient statistical power and can be highly misleading when a meta-analysis includes fewer than 10 pooled studies.

Our findings contrast with some previous reviews that reported stronger associations. This may be because we excluded subjective measures of oral health, which comprised the majority of studies on this topic. Self-reported measures reflect an individual’s perception, which may have a more profound impact on psychological well-being than objective clinical status. Conversely, an individual’s mental state may bias their self-report. It should also be noted that while the heterogeneity in many of our quantitative analyses was very low, this is likely due to the low number of studies in each analysis.

The clinical implications depend on the direction of the association. If oral conditions, particularly tooth loss, precipitate depression, this supports integrating oral health into mental health prevention strategies and ensuring access to dental rehabilitation. Conversely, if depression drives oral disease, mental health screening in dental settings and collaborative care models become priorities.

## Conclusion

The results of this systematic review and meta-analysis reveal a complex, multi-dimensional relationship between clinical oral health conditions and symptoms of depression. Quantitative synthesis provided robust evidence that tooth loss is a risk factor for developing depressive symptoms. Conversely, the presence of depressive symptoms increased the odds of having untreated dental caries, a major cause of tooth loss. Although quantitative analysis was not possible for all conditions in every direction, this study sought to address these gaps and further examine the meta-analysis results through a narrative synthesis. This synthesis indicated that individuals with depression are more likely to experience various oral diseases(dental caries, periodontal diseases, and tooth loss), though the degree and consistency of these associations vary. However, the overall certainty of the evidence remains low, primarily due to the predominance of cross-sectional study designs, which preclude causal inference, and high heterogeneity in exposure and outcome measurements and definitions. Future research should prioritize longitudinal designs with standardized measurements and, ideally, include supplementary analyses using different diagnostic thresholds to facilitate comparison and comprehensive meta-analyses.

## Data Availability

The authors declare that all data generated or analyzed during this study are included in this published article.
